# Multi-Source Generation Mechanisms for Low Frequency Noise Induced by Flood Discharge and Energy Dissipation from a High Dam with a Ski-Jump Type Spillway

**DOI:** 10.3390/ijerph14121482

**Published:** 2017-11-30

**Authors:** Jijian Lian, Xiaoqun Wang, Wenjiao Zhang, Bin Ma, Dongming Liu

**Affiliations:** 1 State Key Laboratory of Hydraulic Engineering Simulation and Safety, Tianjin University, 92 Weijin Road, Nankai District, Tianjin 300072, China; jjlian@tju.edu.cn (J.L.); 1014205052@tju.edu.cn (X.W.); mabin97@tju.edu.cn (B.M.); hpeliudm@tju.edu.cn (D.L.); 2 Yellow River Institute of Hydraulic Research, 46 Shunhe Road, Jinshui District, Zhengzhou 450000, China

**Keywords:** low frequency noise, high dam flood discharge, energy dissipation, ski-jump type spillway, vortex sound model, vorticity fluctuation, nappe-cavity coupled vibration

## Abstract

As excess water is discharged from a high dam, low frequency noise (air pulsation lower than 10 Hz, LFN) is generated and propagated in the surrounding areas, causing environmental hazards such as the vibration of windows and doors and the discomfort of local residents. To study the generation mechanisms and key influencing factors of LFN induced by flood discharge and energy dissipation from a high dam with a ski-jump type spillway, detailed prototype observations and analyses of LFN are carried out. The discharge flow field is simulated and analyzed using a gas-liquid turbulent flow model. The acoustic response characteristics of the air cavity, which is formed between the discharge nappe and dam body, are analyzed using an acoustic numerical model. The multi-sources generation mechanisms are first proposed basing on the prototype observation results, vortex sound model, turbulent flow model and acoustic numerical model. Two kinds of sources of LFN are studied. One comes from the energy dissipation of submerged jets in the plunge pool, the other comes from nappe-cavity coupled vibration. The results of the analyses reveal that the submerged jets in the plunge pool only contribute to an on-site LFN energy of 0–1.0 Hz, and the strong shear layers around the high-velocity submerged jets and wall jet development areas are the main acoustic source regions of LFN in the plunge pool. In addition, the nappe-cavity coupled vibration, which is induced when the discharge nappe vibrates with close frequency to the model frequency of the cavity, can induce on-site LFN energy with wider frequency spectrum energy within 0–4.0 Hz. By contrast, the contribution degrees to LFN energy from two acoustic sources are almost same, while the contribution degree from nappe-cavity coupled vibration is slightly higher.

## 1. Introduction

As a majority of high dams built in recent decades or projected in China have been with the features of high water head, big flood-discharge capacity, deep narrow valley and large flood-discharge power, issues such as energy dissipation and scour protection, vibration control for hydraulic structures, atomization protection and aeration in cavitation protection, have received considerable attention and abundant technological achievements have resulted [1,2,3,4]. Recently, environmental hazards from low frequency noise (LFN) have been found around some hydropower stations in China, as a new research subject in the field of high dam flood discharge studies [5,6]. LFN that has sound pressure level (SPL) significantly larger than background noise has been detected, and doors and windows have been found to vibrate violently during the flood discharge of many hydropower stations in China. For instance, the LFN observed during the flood discharge of the Jinping First Stage Hydropower Station had SPL about 20–50 dB larger than the background noise, and windows and doors of some downstream construction buildings experienced sustained vibration; windows and doors of the ice-making building for the concrete production system of Xiluodu Hydropower Station obviously vibrated; windows of buildings located at the left abutment of the Ertan Hydropower Station oscillated evidently during the flood is discharged. The roller shutter doors of the residential buildings in the downstream village of the Xiangjiaba and Huangjinping Hydropower Stations both experienced sustained vibration during the flood releasing time [5,6].

Japanese scholars have carried out some research on the flow-induced LFN. Because Japan is a densely populated country and many hydropower stations are located closed to residents’ living quarters, the hazards of LFN were first observed in Japan. Most research has been focused on the LFN induced by the oscillating nappe, which is formed when the flood is discharged through a weir, and its adverse impact on the surrounding buildings and inhabitants. The induced LFN can produce squeaking noise of doors and windows in the surrounding areas and cause psychological problems of inhabitants such as insomnia, dysphoria and chest congestion [7,8]. In addition, LFN can travel very far and still be detected thousands of kilometers away from the acoustic source, which makes control or preventive measures difficult to implement. In previous studies, Nakamura [9] reported the phenomenon of LFN induced by flood discharge as early as 1978. Based on their site observations, Takebayashi [10] and Nakagawa [11] carried out further research on the mechanisms of nappe-cavity self-excited oscillation and the characteristics of the LFN. They concluded that the nappe vibrated most violently when the natural vibration frequencies of the nappe and the cavity behind the nappe were almost same. Izumi [12] studied the characteristics of LFN induced by the energy dissipater of hydraulic jumps based on serial model tests. The characteristics of the LFN intensity which were associated with the different hydraulic factors of the dissipater were studied. Ochiai et al. [13] studied the SPL of the noise from the windows and doors induced by LFN by means of series of model and object tests. It was seen that the SPL value was influenced by the frequency of LFN, and fixed conditions of the windows and doors. They regarded the fixed conditions as the key influencing factor. Murakawa et al. [14] studied the control measures of the LFN induced by the weir with automatic platform trap door, and the effects of the measures were estimated based on the on-site observation of the SPL. Gotoh et al. [15] studied the characteristics of the noise of the falling water from the overflow weir with the flow angle of 90° and 30°. All the research indicates that LFN induced by flood discharge can cause environmental hazards to the surrounding areas, and the generation of LFN has close correlation with the flow regime of discharge.

The engineering objects in these past studies have the common characteristics of low water heads, small flow rates and simple flow regimes, and the LFN is induced by relatively continuous nappe. As for the hydraulic projects with high water heads, large flow rates and complex flow regime which dissipate energy by ski-jump type spillways, the previous theories cannot be directly applied. Moreover, LFN energy still can be observed on site in the conditions with single discharge orifice opening or several nonadjacent orifices opening, although the continuous nappe does not exist. Therefore, new research should be done on the generation mechanisms of LFN induced by flood discharge from the high dam by ski-jump type spillways and resulting energy dissipation.

In order to reveal the generation mechanism and key influencing factors of LFN induced by flood discharge and energy dissipation from high dams with ski-jump type spillways, a multi-sources generation mechanism is put forward based on the prototype observation results, vortex sound model, turbulent flow model and acoustic numerical model. Two kinds of sources of LFN are studied. One comes from the energy dissipation of submerged jets in the plunge pool and is studied through a vortex sound model and turbulent flow model, the other comes from nappe-cavity coupled vibration and is studied based on the finite element method coupled boundary element method (FEM/BEM). Analyses on the degree of contribution of the multiple sources to the LFN energy are also made according to the prototype observation results. The results in this study should provide reference data, a theoretical basis and an assessment method for the environmental harm from LFN induced in flood-discharge engineering of high-dams equipped with ski-jump type spillways.

## 2. Materials and Methods

### 2.1. Prototype Observation

#### 2.1.1. Prototype

The Jinping First Stage Hydropower Station is an important controlling cascade built on the downstream main stream portion of the Yalong River. The station consists of a water-retaining structure, flood discharge and energy dissipation structures and a power generation system. The water-retaining structure is a concrete double-curvature arch dam with the maximum height of 305 m, which is the highest in the world [16]. The total installed capacity of this station is 3600 MW. Five diversion bottom outlets, two emptying bottom outlets, five bottom discharge orifices and four crest overflowing orifices are arranged on the dam body. The station began to store water on 30 November 2012 and entered its normal operation phase with a normal water level of 1880 m on 24 August 2014.

#### 2.1.2. Observation System and Conditions

Since Jinping First Stage Hydropower Station began to discharge floodwater, Tianjin University has organized frequent prototype observations of LFN. The on-site LFN observation equipment consists of an infrasound microphone, multi-channel digitizer and computer. The infrasound microphone operates in the frequency range of 0.1–300 Hz with a sensitivity of 150 mv/Pa. The multi-channel digitizer connects the microphone with the computer, which supplies power to the microphone and saves the data at high speed. The digitizer has a built-in GPS, so it can work well in the open air.

**Figure 1:**
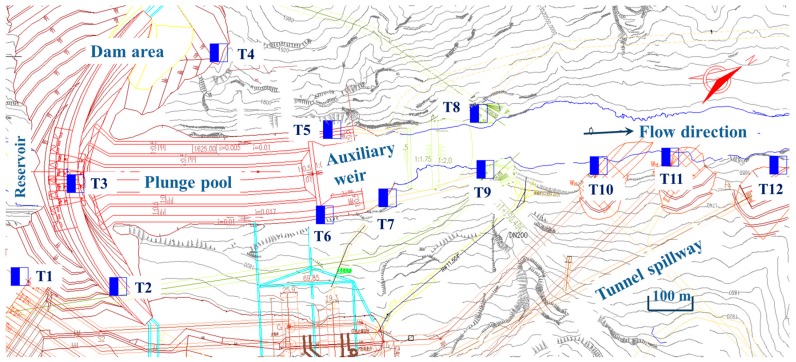
Observation points arrangement of LFN.

The observation points are distributed along the river valley from the dam area to the downstream location which is approximately 1.25 km away from the dam body. The number of observation points is 12 in total (T1–T12 in Figure 1). The observation work has been carried out at least twice at the same point under the same discharge conditions. The sampling time of the observed LFN data is set as 3–6 min. The sampling frequency is set as 600 Hz. Table 1 shows the details of the observed discharge conditions.

### 2.2. Theoretical Model of Vortex Sound

In the hydraulic projects with ski-jump type spillways to discharge flood, the enormous kinetic energy carried by the discharged nappe is dissipated in the plunge pool. According to the turbulent jet theory, the flow field along the direction of the entering nappe jets in the plunge pool can be generally divided into three regions, including the submerged jet region, impact region and wall jet region. The large flow velocity gradient results in numerous low-frequency large coherent structures generated continuously in the strong shear layers around the high-velocity submerged jets. The development of the shear layers is dominated by the interaction between the large-scale vortex structures, such as the coalescence, pairing and entrainment [17]. The vortex structures in the wall jet region have been studied by Kline et al. [18]. Various vortexes with different scale and strength have been found in the wall jet region, and the slanted horseshoe vortex or hairpin-shape vortex is most common.

**Figure 2:**
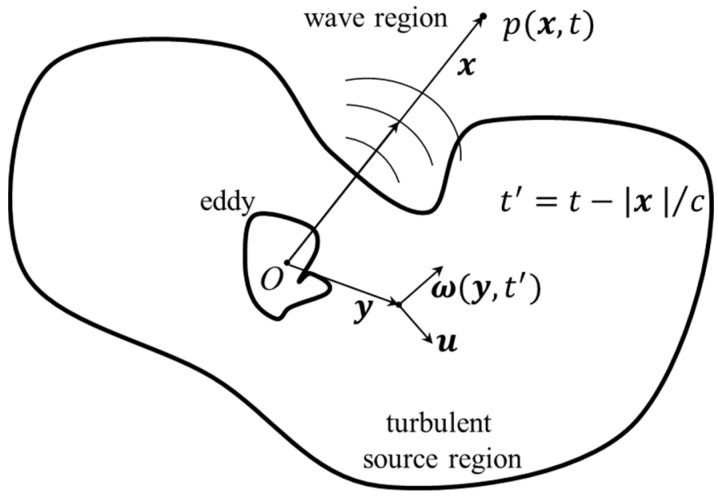
Sketch map of the vortex sound theory.

In the acoustic area, Powell [19] and Howe [20,21] et al. have studied the basic issues of the internal mechanism of fluid vocalization and the interaction between acoustic wave and turbulent flow in terms of vortex dynamic theory since 1960s. They have established the vortex sound theory, and proposed that the vortex is a significant acoustic source. The vortex sound theory is feasible and effective to identify the generation mechanism of flow-induced noise at a low Mach number. In the turbulent flow field of flood discharge and energy dissipation with the ski-jump type spillway from a high dam, various vortices always exist, so the formation and transition of acoustic energy, which comes from the interaction of vortical structures, potential flow and solid boundaries, cannot be neglected. Therefore, the vortex sound theory is carried out on the mechanism study of LFN induced by ski-jump energy dissipation in this paper. Assuming that the flow is incompressible and isentropic, and basing on the equations of continuity and motion, the vortex sound equation can be derived and simplified as [19]:

(1) $$\nabla^{2}p-\frac{1}{c^{2}}\frac{\partial^{2}p}{\partial t^{2}}=-q,$$

where p is a quantity which agrees in the far field with the acoustic pressure fluctuations; c is the ambient speed of sound; q is a source term which can be calculated from the flow, which can be taken as:

(2) q=ρ div L, L=ω×u,

where ω=curl u denotes the vorticity vector in which u is flow velocity vector; ρ is ambient density. The vortex sound equation is a typical nonhomogeneous wave equation, with the differential expression of the propagation process of the acoustic wave in a nonhomogeneous fluid on the left hand and the acoustic source term of the vortex on the right hand. Equation (1) indicates that the flow-induced acoustic pressure is directly related to the sizes and motion of the vortices. For an example refers to an unsteady low Mach number flow in free space, the general solution of Equation (1) for the far field is expressed as follows using the Green function method [22]:

(3) $$p\left(x,t\right)=\rho \frac{\partial }{\partial t^{\prime }}\int \;G\left(x,t;y,t^{\prime }\right)\cdot \omega \left(y,t^{\prime }\right)d^{3}ydt^{\prime },$$

where, as shown in Figure 2, x is a displacement vector in the wave region and y is a displacement vevtor in the flow region; |x|=x and |y|=y; t denotes time of observation and $t^{\prime }=t-x/c$ is a retarded time for wave propagation; p(x,t) is a solution of the p in Equation (1) on the point x at time t. Meanwhile the vector Green function must satisfy $\nabla_{y}G\left(x,t;y,t^{\prime }\right)=\nabla_{y}\times \nabla_{y}G\left(x,t;y,t^{\prime }\right)$.

The integrand on the right of Equation (3) only contain the vorticity variable ω, manifesting that the region with time-varying vorticity is the effective acoustic source region. Furthermore, since the flow velocity is not contained in the integrand, the acoustic pressure in the wave region can be calculated through effective vorticity fluctuation data directly. As G is a symmetric function, according to Powell’s deduction [19], G can be confirmed as:

(4) $$G\left(x,t,y,t^{\prime }\right)=\frac{1}{12\pi c^{2}x^{3}}\delta^{\prime \prime }\left(t-t^{\prime }-\frac{x}{c}\right)\left(x\cdot y\right)x\times y,$$

where δ is Dirac delta function. Then Equation (3) leads to:

(5) $$p\left(x,t\right)=\frac{\rho }{12\pi c^{2}x^{3}}\rho \frac{\partial^{3}}{\partial t^{3}}\int \left(x\cdot y\right)x\times y\cdot \omega d^{3}y,$$

where $\omega =\omega \left(y,t^{\prime }\right)$ in the flow region depends on the retarded time $t^{\prime }=t-x/c$. As the attenuation of acoustic pressure during propagation is not included above, a sonar equation is built as:

(6) $$SL-TL=20\log_{10}\frac{P_{e}}{P_{ref}}-{\left(\int \alpha dL\right)}_{y}=20\log_{10}\frac{P^{\prime }}{P_{ref}}$$

where *SL* is the RMS of SPL without regard to attenuation; *TL* is about SPL attenuation; $P_{e}$ is the RMS of acoustic pressure calculated by Equation (5); $P_{ref}$ is the reference value of acoustic pressure, set as $2\times 10^{-5}$ Pa; α is SPL attenuation rate; $P^{\prime }$ is the virtual value of acoustic pressure finally achieved with regard to attenuation. The mathematical prediction model of LFN in this paper is based on the Equations (5) and (6), and the LFN intensity can be predicted by substituting the results of numerical simulation of the near-field turbulent flow into the prediction model.

### 2.3. Acoustic Numerical Model of Nappe-Cavity

In the acoustic theory, the acoustic wave equation can be expressed as:

(7) $$\nabla^{2}p\left(x,t\right)-\frac{1}{c^{2}}\frac{\partial^{2}p\left(x,t\right)}{\partial t^{2}}=0,$$

The acoustic Helmholtz equation can be deduced from taking the Fourier transform of the Equation (7) and be expressed as:

(8) $$\nabla^{2}p\left(x\right)-k^{2}p\left(x\right)=0,$$

where $p\left(x,t\right)=p\left(x\right)e^{i\omega t}$; k=ω/c is wave number, ω is angular frequency. The Helmholtz equation can be solved by FEM/BEM method.

When using the acoustic FEM, the acoustic matrix equation for finite element analysis can be written as:

(9) $$\left(K_{a}-\omega^{2}M_{a}\right)\cdot \left\{P_{i}\right\}=\left\{F_{ai}\right\},$$

where $K_{a}$ is the acoustic stiffness matrix; $M_{a}$ is the acoustic mass matrix; $\left\{P_{i}\right\}$ is the sound pressure matrix; $\left\{F_{ai}\right\}$ denotes the acoustic excitation and the subscript ‘*i*’ in the equation denotes the *i*-th node and the subscript ‘*a*’ indicates that the items in the equation is about acoustic system.

Acoustic mode is a unique property of the air medium [23,24], which is mainly related to the shape and location of the air cavity. Because the air cavity is a vibration system with its mass and compressibility, it has a series of modal frequencies and modal shapes. When a periodic exciting force acts on the cavity and the exciting frequency coincides with certain natural frequency of the cavity, the acoustic resonance occurs. The characteristic parameters of cavity acoustic modes, which are the eigenvalues of the acoustic mass and stiffness matrixes in Equation (9), can be calculated by the acoustic FEM. Then, Equation (7) leads to:

(10) $$\left(K_{a}-\omega^{2}M_{a}\right)\left\{\Phi \right\}=0,$$

where {Φ} is the acoustic mode matrix. In a linear space, an arbitrary acoustic pressure distribution matrix can be linearly expressed by several acoustic modal characteristic matrixes in Equation (10):

(11) $$\left\{P_{i}\right\}=\lambda_{1}\Phi_{1}+\lambda_{2}\Phi_{2}+\ldots +\lambda_{n}\Phi_{n}=\left\{\Phi \right\}\cdot \lambda ,$$

where $\left\{\Phi \right\}=\left(\Phi_{1},\;\Phi_{2},\ldots ,\Phi_{n}\right)$ and $\Phi_{i},\;i=1,\;2,\;\ldots ,\;n$ is the *i*-th order acoustic modal shape of the cavity; $\lambda ={\left(\lambda_{1},\lambda_{2},\ldots ,\lambda_{n}\right)}^{T}$ and $\lambda_{i}$ is the acoustic modal participation factor (AMPF) of $\Phi_{i}$, which represents the contribution degrees from every order acoustic mode to the acoustic response in the cavity. The AMPF can be calculated by the linear superposition theory or residue method.

Furthermore, the acoustic direct BEM can be used to calculate the acoustic field distribution in the cavity [25]. The velocity boundary is chosen as the acoustic boundary condition of the cavity. On the velocity boundary $\Omega_{v}$, the solution of Equation (6) should satisfy:

(12) $${\bar{v}}_{n}\left(r\right)=\frac{j}{\rho_{0}\omega }\frac{\partial p\left(r\right)}{\partial n},\;r\in \Omega_{v},$$

where n is the normal vector of the boundary; $\rho_{0}$ is air density in cavity and ${\bar{v}}_{n}\left(r\right)$ is the normal velocity on $\Omega_{v}$. For solving the closed equations of Equations (8) and (12), all of the acoustic boundaries $\Omega_{a}$ should be dispersed into several elements and nodes first, and then the acoustic pressure $p\left(r_{a}\right)$ and normal velocity $v_{n}\left(r_{a}\right)$ of an arbitrary point belonging to every boundary element can be expressed as:

(13) $$\{\begin{array}{c} p\left(r_{a}\right)=\overset{n_{a}}{\underset{i=1}{\sum }}N_{i}\cdot p_{i}=N_{i}\left\{p_{i}\right\},\;r_{a}\in \Omega_{a}\\ v_{n}\left(r_{a}\right)=\overset{n_{a}}{\underset{i=1}{\sum }}N_{i}\cdot v_{ni}=N_{i}\left\{v_{ni}\right\},\;r_{a}\in \Omega_{a} \end{array},$$

where $N_{i}$ is the global shape function and $n_{a}$ is the number of nodes on the whole boundary element grids. To any node b on the boundary element grids in the cavity, the acoustic pressure can be obtained as:

(14) $$A_{b}\left\{p_{i}\right\}=j\rho_{0}\omega B_{b}\left\{v_{ni}\right\},\;b=1,2,\ldots n_{a},$$

where $A_{bi}=\delta_{bi}\left[1+\frac{1}{4\pi }\int \frac{\partial }{\partial n}\left(\frac{1}{\left|r_{a}-r_{b}\right|}\right)d\Omega \left(r_{a}\right)\right]-\int N_{i}\left(r_{a}\right)\frac{\partial G\left(r_{b},r_{a}\right)}{\partial n}{d\Omega }_{a}$; $B_{bi}=\int N_{i}\left(r_{a}\right)G\left(r_{b},r_{a}\right){d\Omega }_{a}$; $\delta_{bi}$ is the Kronecker function and $G\left(r_{b},r_{a}\right)$ is the Green function. To any node which is not on the boundary element grids and has the distance vector r, the acoustic pressure can be obtained as:

(15) $$p\left(r\right)=C^{T}\left\{p_{i}\right\}+D^{T}\left\{v_{ni}\right\},\;r\in V,\;r\notin \Omega_{a},$$

where $C_{i}=\int N_{i}\left(r_{a}\right)\frac{\partial G\left(r,r_{a}\right)}{\partial n}{d\Omega }_{a},\;i=1,\;2,\;\ldots ,n_{a}$, $D_{i}=j\rho_{0}\omega \int N_{i}\left(r_{a}\right)G\left(r,r_{a}\right){d\Omega }_{a},\;i=1,\;2,\;\ldots ,\;n_{a}$.

### 2.4. Numerical Turbulent Flow Model

#### 2.4.1. Gas-Liquid Turbulent Model

Past studies have indicated that the k−ε turbulent flow model is a robust method for simulating the hydrodynamic characteristics of turbulent flow [26,27]. Its improved version, the RNG k−ε model, first developed by Yakhot and Orszag [28], can simulate flow with high strain rate and large streamline curvature better than the traditional k−ε model. Therefore, the RNG k−ε turbulent flow model is employed. The VOF method, put forward by Hirt and Nichols [29] in 1975 based on the MAC method, is an effective way to deal with the complex free water surface [30]. So the VOF method is employed in this study to deal with gas-liquid two phase problem. The major governing equations are listed below:

Basic equations of turbulent flow:

(16) $$\{\begin{array}{c} \frac{\partial \rho }{\partial t}+\frac{\partial \rho u_{i}}{\partial x_{i}}=0\\ \frac{\partial u_{i}}{\partial t}+\frac{\partial u_{i}u_{j}}{\partial x_{j}}=-\frac{1}{\rho }\frac{\partial p}{\partial x_{i}}+\frac{1}{\rho }\frac{\partial }{\partial x_{j}}\left[\left(\mu +C_{\mu }\frac{k^{2}}{\varepsilon }\right)\left(\frac{\partial u_{i}}{\partial x_{j}}+\frac{\partial u_{j}}{\partial x_{j}}\right)\right] \end{array},$$

k equation:

(17) $$\frac{\partial \left(\rho k\right)}{\partial t}+\frac{\partial \left(\rho ku_{i}\right)}{\partial x_{i}}=\frac{\partial }{\partial x_{j}}\left[\alpha_{k}\mu_{eff}\frac{\partial k}{\partial x_{j}}\right]+G_{k}+\rho \varepsilon ,$$

ε equation:

(18) $$\frac{\partial \left(\rho \varepsilon \right)}{\partial t}+\frac{\partial \left(\rho \varepsilon u_{i}\right)}{\partial x_{i}}=\frac{\partial }{\partial x_{j}}\left[\alpha_{\varepsilon }\mu_{eff}\frac{\partial \varepsilon }{\partial x_{j}}\right]+\frac{C_{1\varepsilon }^{*}}{k}G_{k}-C_{2\varepsilon }\rho \frac{\varepsilon^{2}}{k},$$

Gas-liquid VOF equation:

(19) $$\{\begin{array}{c} \alpha_{a}=1-\alpha_{w}\\ \frac{\partial \alpha_{w}}{\partial t}+u_{i}\frac{\partial \alpha_{w}}{\partial x_{i}}=0 \end{array},$$

where $\mu_{eff}=\mu +\rho C_{\mu }\frac{k^{2}}{\varepsilon }$, $C_{1\varepsilon }^{*}=C_{1\varepsilon }-\frac{\eta \left(1-\eta /\eta_{0}\right)}{1+\beta \eta^{3}}$, $\eta ={\left(2E_{ij}\cdot E_{ij}\right)}^{1/2}\frac{k}{\varepsilon }$, $E_{ij}=\frac{1}{2}\left(\frac{\partial u_{i}}{\partial x_{j}}+\frac{\partial u_{j}}{\partial x_{j}}\right)$, $C_{\mu }=0.0845$, $\alpha_{k}=\alpha_{\varepsilon }=1.39$, $C_{1\varepsilon }=1.42$, $C_{2\varepsilon }=1.68$, $\eta_{0}=4.377$, β=0.012, and $\alpha_{w}$ and $\alpha_{a}$ are the volume fractions of water and air in a unit grid, respectively.

#### 2.4.2. Simulation Domain and Boundary Conditions

**Figure 3:**
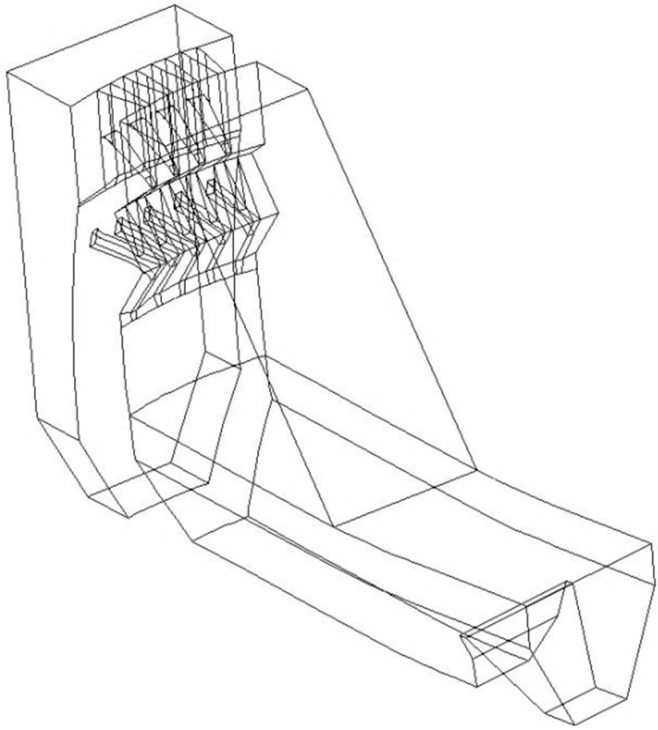
Numerical turbulent flow model of Jinping.

The numerical turbulent flow model established is a 1:1 scale simplified arch dam of Jingping, as shown in Figure 3. The conditions calculated have been shown in Table 1. The simulation domain includes the water body extended 50-m into the upstream reservoir, the arch dam segments with the bottom orifices and crest overflowing orifices, the discharge nappe area in the air, the plunge pool and the water body 40-m long after the tail-weir. The model is meshed by block-structured grids with the grid size 2 m long × 1 m wide × 1 m high. For the main flood discharge and energy dissipation regions, such as the bottom discharge orifices, the crest overflowing orifices, the falling nappe area in the air and the first half of the plunge pool, the grid size is set as 1 m long × 1 m wide × 1 m high, and the mesh refinement method adaptive to the variations of the pressure gradient and free water surface is employed during the calculation. The total number of grid units is approximately 2 million. The finite volume method is employed to build the discrete equations. The PISO method is employed to do the pressure-velocity coupled calculation. The wall function method is used to calculate the near-wall flow field.

As for the boundary conditions, the upstream inlet of the reservoir and the downstream outlet of the plunge pool are set as the pressure inlet and pressure outlet, respectively. The top boundaries of the model and the both sides of nappe area are set as air pressure inlets with the normal atmospheric pressure. Other boundaries are set as no-slip wall. The time step size of unsteady flow calculation is set to 0.001 s. The calculation is considered to be completed when the deviation of mass flow rates between inlet and outlet is less than 0.1%.

## 3. Results and Discussion 

The analyses of the LFN generation mechanisms have been separated into three parts. The multi-sources mechanisms are introduced basing on the prototype observation. Then two kinds of mechanisms have been analyzed. One comes from energy dissipating submerged jets in the plunge pool and is studied through vortex sound model and turbulent flow model, the other comes from nappe-cavity coupled vibration and is studied based on finite element method coupled boundary element method (FEM/BEM).

### 3.1. Prototype Observation

**Figure 4:**
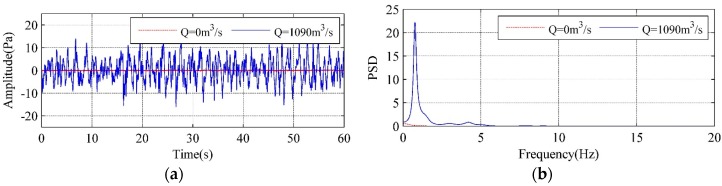
Comparisons of the time history and PSD curves of LFN: (**a**) time history curves; (**b**) power spectral density curves.

Figure 4 compares the time history and power spectral density (PSD) curves of the LFN observed at T11 between the no-releasing discharge condition (Condition 1) and Condition 2 in Table 1. It can be seen that the LFN intensity greatly increases during the flood discharge, and the acoustic energy mainly concentrated within the frequency domain of 10 Hz. It is obvious that LFN is induced in the process of flood discharge.

#### 3.1.1. Spatial Distribution and Propagation Patterns

**Figure 5:**
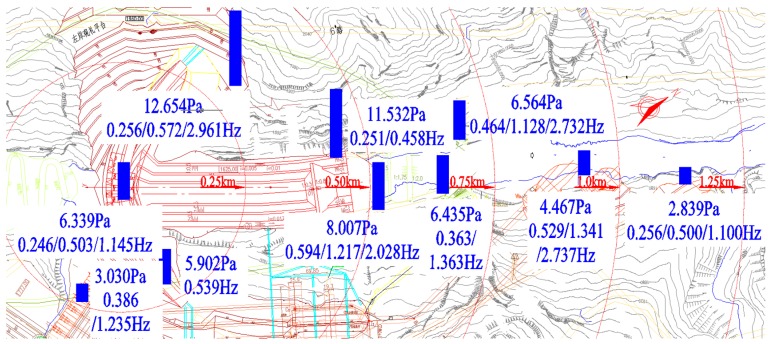
Numerical turbulent flow model of Jinping.

**Figure 6:**
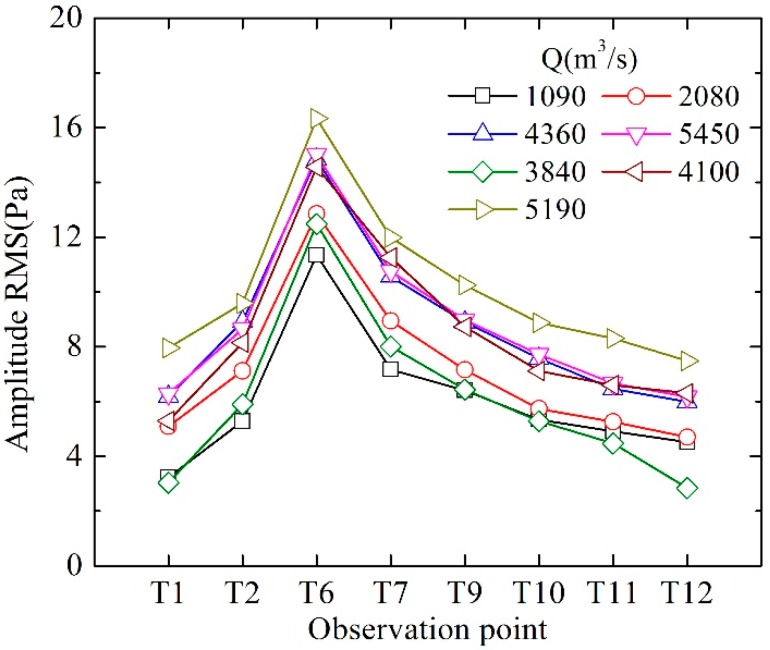
LFN amplitude distribution along the downstream river valley.

The spatial distribution of the LFN along the downstream area is analyzed. Figure 5 shows the spatial distribution of the LFN amplitude observed in Condition 6. The LFN amplitude of eight observation points located along the right bank side (T1, T2, T6, T7, T9, T10, T11, and T12) has been measured and shown in Figure 6. It gets the maximum near the energy dissipation area (T6) in the plunge pool. The amplitude of LFN decreases gradually with increasing distance away from the energy dissipation area. According to the calculation of statistical average, the LFN intensity in the flood discharge and energy dissipation area (within a range of 500 m downstream from the dam body) attenuates at a rate of approximately 0.0411 Pa/m (0.0311 dB/m), which decreased to 0.00586 Pa/m (0.00716 dB/m) at a range between 500 m and 1250 m downstream from the dam body.

#### 3.1.2. Correlation between LFN and Discharge Flow Regime

The relation between the observed LFN intensity and flood discharge capacity is analyzed. The amplitude of LFN is found to not increase in direct proportion to Q. A parameter is set as P/Q for further analyses, where P is the root mean square (RMS) of the LFN amplitude. Table 2 lists the values of Q, P and P/Q of several observation points. It is seen that P/Q gets the minimum under Condition 5 and Condition 6. The maximum of P/Q is found in Condition 2, which are approximately three or four times larger than the minimum. In the conditions of flood discharged by the bottom orifices (Conditions 2–5), the values of P/Q decrease gradually with the increasing number of opening orifices and Q. The conditions with more adjacent orifices opening uniformly are found effective to reduce the LFN intensity, because such discharge operation regimes can avoid flow concentrated in the plunge pool, which means maintaining smooth and uniform flow regime may reduce the LFN intensity.

**Figure 7:**
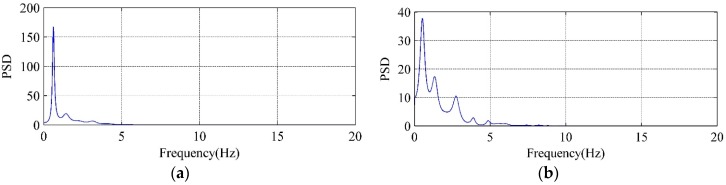
Comparisons of the PSD curves of LFN observed at T9: (**a**) Condition 2; (**b**) Condition 6.

The PSD curves of LFN are calculated to analyze the frequency-domain characteristics. It is found that LFN data observed with different flow regimes has different characteristics. Figure 7 displays the PSD curves of LFN observed at T9 under Condition 2 and Condition 6. To the conditions with the nonadjacent orifices opening (e.g., Condition 2), the observed LFN has single dominant frequency which concentrates within 1 Hz. To the conditions with the adjacent orifices opening (e.g., Condition 6), an air cavity is formed between the discharge nappe and dam body, and the observed LFN has wider energy distribution and several peak frequencies within 4 Hz. It reveals that multi-sources generation mechanisms make contribution to induce the LFN during the flood discharge of Jinping First Stage Hydropower Station. This summary will be specifically studied and identified in the following section.

### 3.2. Generation Mechanism for LFN Induced by Submerged Jets

The results of prototype observation indicate that LFN is induced during flood discharge and multi-sources generation mechanisms make contribution to induce the LFN. In this study, we propose that the LFN are induced by two kinds of sources. One comes from energy dissipation of submerged jets in the plunge pool, the other comes from nappe-cavity coupled vibration. In this section, the former source will be analyzed first.

#### 3.2.1. Validation of the Calculation of Turbulent Flow

The calculation of turbulent flow is validated by the flow regime, nappe trajectory distance and water-entry velocity. Due to the space limitations, only the numerical results of several typical discharge conditions are shown, such as Conditions 2 and 6.

**Figure 8:**
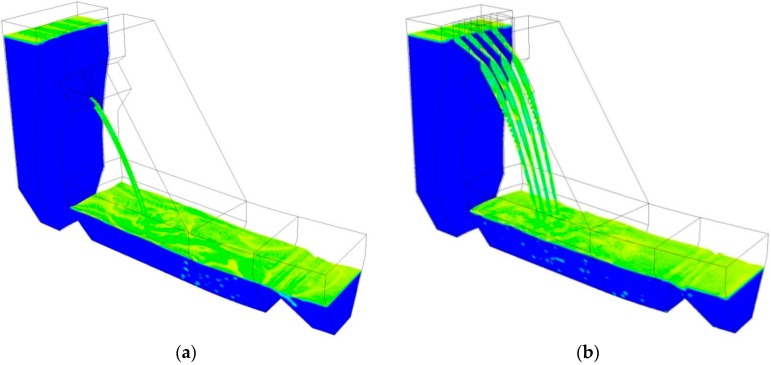
Flow regime of the numerical calculation region: (**a**) Condition 2; (**b**) Condition 6.

In this study, free surface of water in the flow field is determined by air volume fraction of 0.6. Figure 8 shows the flow regime of the whole calculation region of Conditions 2 and 6. As the VOF method is based on the assumption of no-rupturing and no-mixing between air and water, it is difficult to simulate the diffusion of the nappe. As a result, the free surface of the nappe in the numerical results is slightly different with the actual situation. However, the general track and form of the movement of nappes in the air are coincided with physical truth.

The trajectory distance and water-entry velocity of the discharge nappe are the key parameters in the safety problem for hydraulic structures. The theoretical equation for nappe trajectory distance, which has considered internal turbulent diffusion, aeration and the influence of air resistance [31], can be expressed as follows:

(20) $$L=L_{1}\left(1-\frac{1}{6}\frac{C_{f}}{\beta_{0}}\frac{F_{r0}^{2}}{\sin\theta_{0}}\xi^{2}\frac{1-\xi \sin\theta_{0}+0.1\xi^{2}}{\sqrt{1+\frac{2}{F_{r0}^{2}\sin^{2}\theta_{0}}\frac{\Delta S}{H_{0}}}}\right),$$

and:

(21) $$\{\begin{array}{c} L_{1}=\frac{u_{0}^{2}\sin2\theta_{0}}{2g}+\left(1+\sqrt{1+\frac{2g\Delta S}{u_{0}^{2}\sin^{2}\theta_{0}}}\right)\\ F_{r0}=\frac{u_{0}}{\sqrt{gH_{0}}}\\ \xi =\frac{L_{1}}{H_{0}}\frac{1}{F_{r0}^{2}\cos\theta_{0}} \end{array},$$

where $L_{1}$ is the nappe trajectory distance calculated by free parabolic theory; ΔS is the height difference between water surface of plunge pool and the end of flip bucket; $F_{r0}$ is the Froude number of nappe jets; ξ is the dimensionless quantity; $u_{0}$, $\beta_{0}$, $H_{0}$ are the mean velocity, mean water concentration and half-breadth of the nappe section on the end of flip bucket respectively. In Equation (20), for deflecting flow, $\theta_{0}>0$, and $L<L_{1}$, and for dropping flow, $\theta_{0}<0$, and $L>L_{1}$. In addition, the water-entry velocity of nappe jets can be calculated by the equation of the free projectile theory:

(22) $$U=\sqrt{2g\Delta S+u_{0}^{2}},$$

In actual calculation, the bucket angles ($\theta_{0}$) of 3# crest overflowing orifice and 3# bottom discharge orifice are −41.67° and −45.00°, respectively. Table 3 lists some comparisons of numerical and the theoretical results of nappe trajectory length and water-entry velocity. It can be seen that the theoretical results of nappe trajectory length are slightly larger than numerical results, as the theoretical equation considered the aeration and spallation of the jet. The numerical results of water-entry velocity are less than the results of free projectile theory, as the theory has not considered the influence of air resistance. Nevertheless, the relative error between numerical and theoretical results is less than 3.5%. It validates the accuracy of the numerical simulation results, and lays the foundation for further analysis.

#### 3.2.2. Flow Velocity Distribution

**Figure 9:**
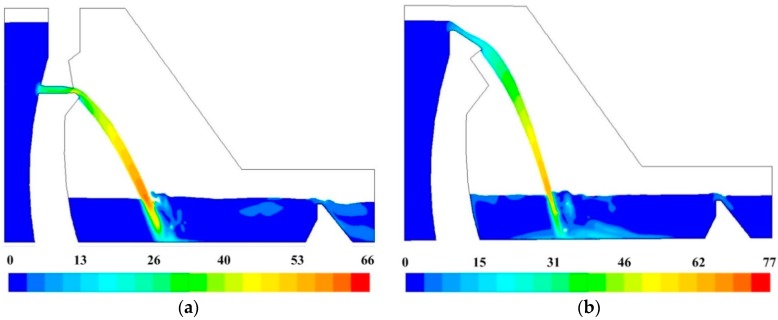
Velocity contours of the surface and deep outlet’s mid-line sections (the unit is m/s): (**a**) Condition 2; (**b**) Condition 6.

The turbulent flow model has been validated in Section 3.2.1. The distribution of the flow velocity is analyzed in this section. Figure 9 shows the velocity contours of the mid-line sections of the orifices. It can be seen that the velocity increase gradually under the action of gravity after the nappe flow out the orifices, and the streamline bends downwards gradually. It reaches maximum about 60 m/s when the nappe arrives on the water surface of the plunge pool. Then the nappe turns to high-speed submerged jet. In the submerged jet region, the jet follows the rules of linear diffusion. The great velocity gradient induces strong turbulent shear region on the junction of high-velocity submerged jets and macroscopical vortex region. The velocity of the jet decays rapidly and drops to 5–14 m/s before the jet arriving at the floor of plunge pool. In the impact region, the main jet is separated into two wall jets by the wall and turn to steady flow before subsidiary dam. In conclusion, the energy dissipation of the downstream water in plunge pool mainly occurs in strong turbulent shear layers.

#### 3.2.3. Correlation Analysis for the Acoustic Source

**Figure 10:**
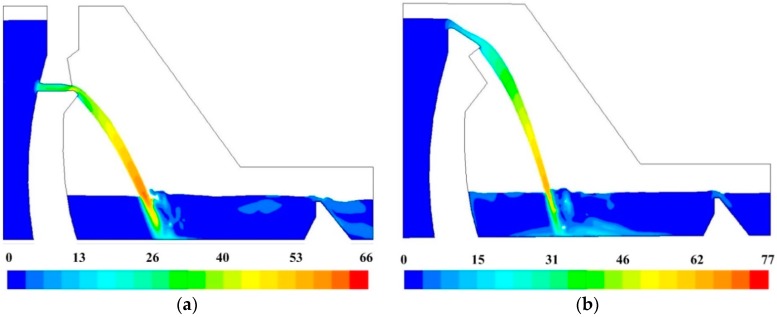
Vorticity contours in energy dissipation area (the unit is s^−1^): (**a**) Condition 2; (**b**) Condition 6.

**Figure 11:**
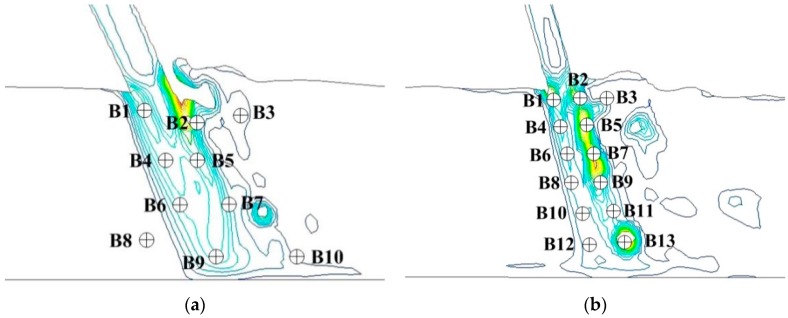
Vorticity monitoring points of mid-line sections of surface and deep orifices: (**a**) Condition 2; (**b**) Condition 6.

According to the vortex sound model, the radiated acoustic field is directly related to the vorticity fluctuation features (VFF). The VFF of energy dissipation field should be analyzed. The correlation between vortex motion and far-field sound pressure can be revealed and the location of effective acoustic source region of the LFN can be estimated by analyzing VFF. Figure 10 shows the vorticity contours of mid-line sections of the orifices. Combining Figure 9 and Figure 10, the vorticity fluctuation is found strongest in the strong shear layers of submerged jets region throughout the whole energy dissipation field. Therefore, many monitoring points have been set on submerged jets region in numerical model to analyze the VFF in the energy dissipation field, as it can be seen in Figure 11. The monitoring time interval is set as 0.005 s. According to the theorem of Nyquist Sampling, the highest analyzing frequency of vorticity monitoring data is 100 Hz, which meets the analysis requirements.

The VFF data have been extracted from numerical simulation and analyzed in time and frequency domain. Table 4 has list the time-average values, RMS values and dominant frequency of vorticity fluctuation of every monitoring point. The vorticity of two areas have been found fluctuating most intensely. One is the strong shear layers around the high-velocity submerged jets in the hydraulic jump, and the other is the wall jets development area. The dominant frequency of vorticity fluctuation distributes within 0–1 Hz.

**Figure 12:**
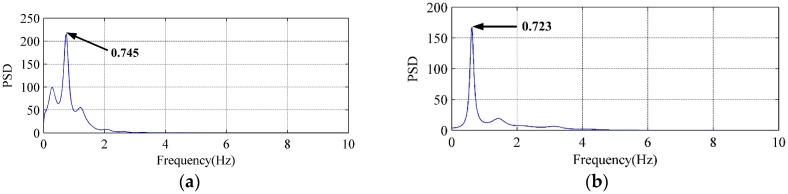
Comparison between vorticity monitoring data and prototype observation data of Condition 2: (**a**) Vorticity monitoring point B2; (**b**) LFN prototype observation point T11.

**Figure 13:**
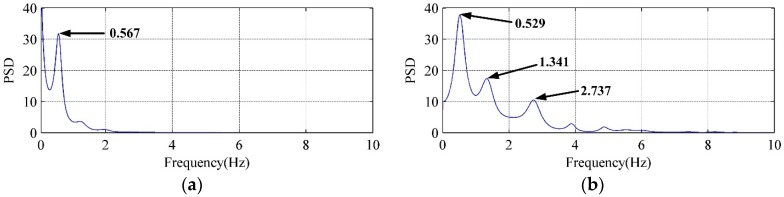
Comparison between vorticity monitoring data and prototype observation data of Condition 6: (**a**) Vorticity monitoring point B5; (**b**) LFN prototype observation point T9.

The PSD curves in Figure 12 and Figure 13 have compared the VFF data of typical vorticity monitoring points with the LFN data from prototype observation. It can be seen that the peak frequencies are very close (within 0–1 Hz), which means that the vorticity fluctuation in energy dissipation field has great relation to the LFN observed in prototype test. Nevertheless, several peak frequencies larger than 1 Hz in the prototype observation result in Figure 13, which is observed in the continuous orifices opening condition (Condition 6), suggest that mechanisms different from the submerged jets must have contribution to the LFN generation. As for the submerged jets in plunge pool, it only contribute to 0–1.0 Hz LFN energy on site, and the strong shear layers around high-velocity submerged jets and wall jet development area are the main acoustic source regions of LFN in plunge pool.

### 3.3. Generation Mechanism of LFN Induced by Nappe-Cavity Coupled Vibration

**Figure 14:**
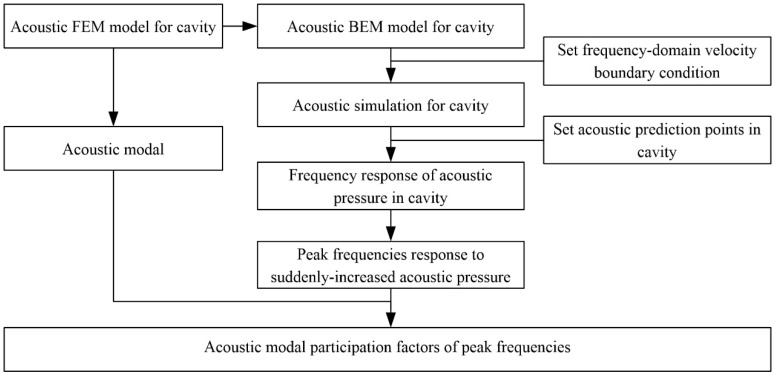
Flowchart of acoustic simulation calculation for cavity.

The analyses in Section 3.2 indicate that the submerged jets in plunge pool only contribute to 0–1 Hz LFN energy on site, and mechanisms different with the submerged jets must contribute to the LFN larger than 1.0 Hz. The results of prototype observation in Section 3.1 indicate that the LFN with peak frequency larger than 1.0 Hz can be observed only in the conditions with the adjacent orifices opening such as Condition 6, in which conditions the air cavity is formed between the discharged nappe and dam body. In this section, the LFN induced by nappe-cavity coupled vibration will be studied through the method of FEM/BEM to simulate the acoustic field in the cavity behind the nappe jets. FEM is employed to calculate the acoustic modal of the cavity, while BEM is employed to analyze the response characteristics of cavity sound field. Figure 14 is the flowchart of acoustic simulation for cavity. Detailed analyses will presented only basing on condition 6 due to the space limitation.

#### 3.3.1. Analysis of Acoustic Modal of the Cavity behind the Nappe Jets

**Figure 15:**
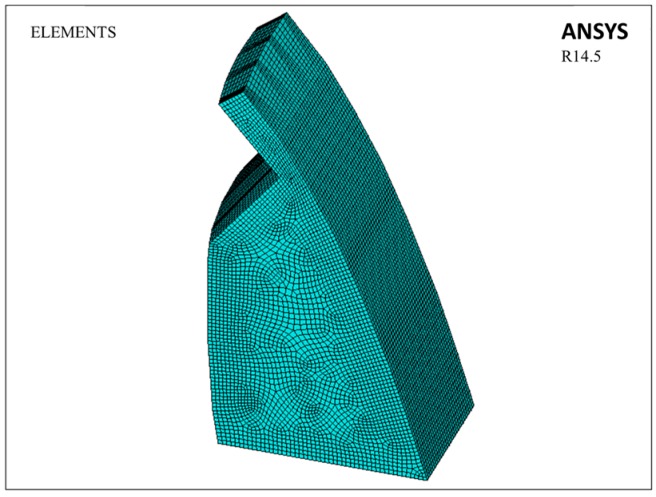
Cavity model of Condition 6.

**Figure 16:**
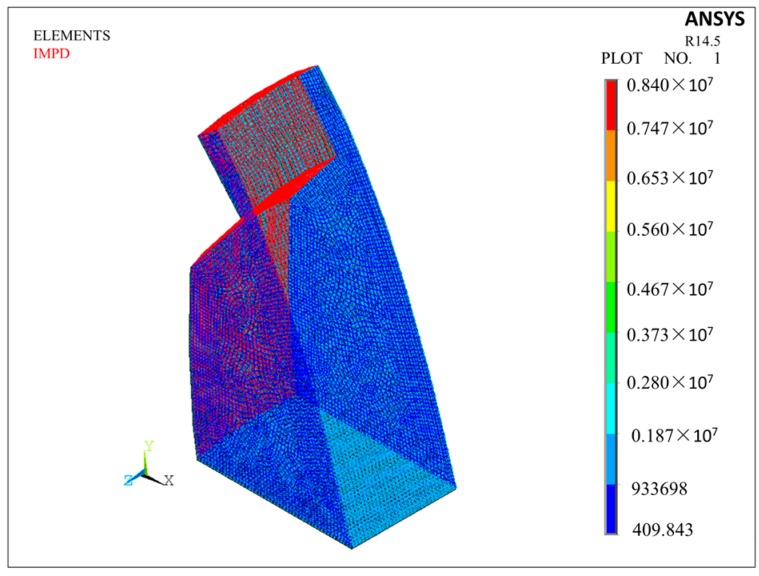
Boundary condition of model.

The 3D finite element model of the cavity behind the nappe jets in Condition 6 has been established through ANSYS software, basing on the cavity shape simulated in Section 3.2. Figure 15 shows the cavity model and grid division result. According to the climate of prototype observation, temperature is set as 25 °C, air density ($\rho_{a}$) 1.1839 kg/m^3^, and the velocity of sound ($C_{0}$) in air 346.18 m/s. Both sides of the cavity are directly connected to the atmosphere, so the acoustic wave can directly transmit to the atmosphere. Therefore, the acoustic impedance of the boundaries, which is defined as the ratio of acoustic pressure on wave-amplitude plane and particle velocity going through this plane, should be defined in this model. The boundary condition can be seen in Figure 16. Air acoustic impedance is added to both sides of the cavity, concrete acoustic impedance is added to the boundary face of arch dam, and water acoustic impedance is added to the boundary of water surface in plunge pool and the continuous nappe jets. The unsymmetric method is employed to calculate and analyze the acoustic modal. Table 5 lists the results of the natural frequency of the cavity in first 10 order.

**Figure 17:**
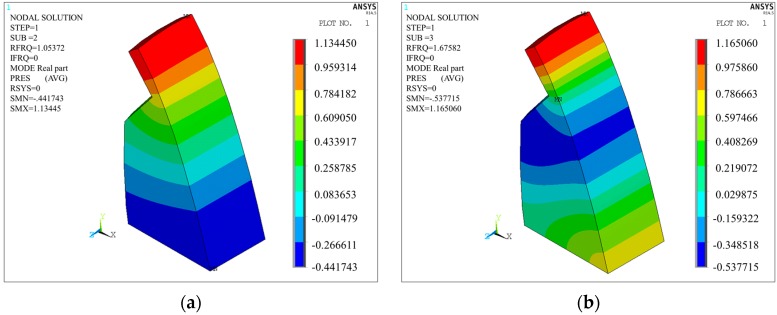

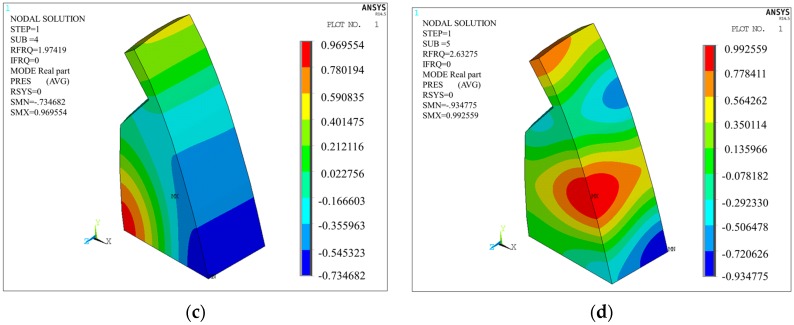
Cavity acoustic modal vibration mode of Condition 6: (**a**) Second order; (**b**) Third order; (**c**) Fourth order; (**d**) Fifth order.

The modal frequency of first order is 0, which is the rigid modal. According to Table 5, the first 10 order modal of the cavity have frequencies within 0–4 Hz. Figure 17 shows the pressure contours of the cavity from the second order to the fifth.

#### 3.3.2. Acoustic Response Analysis

**Figure 18:**
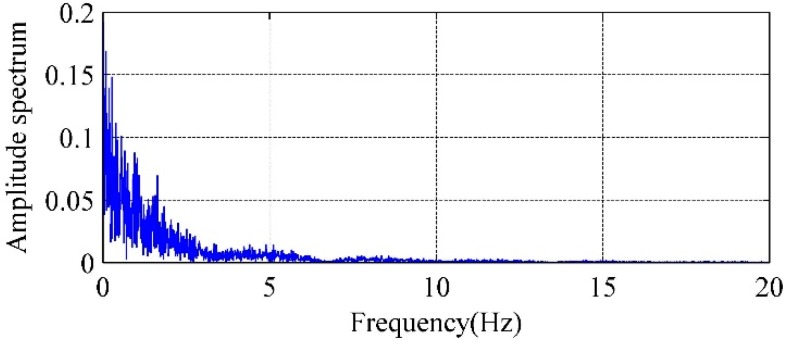
Amplitude spectrum of the longitudinal flow velocity of 3# crest overflowing orifice outlet.

**Figure 19:**
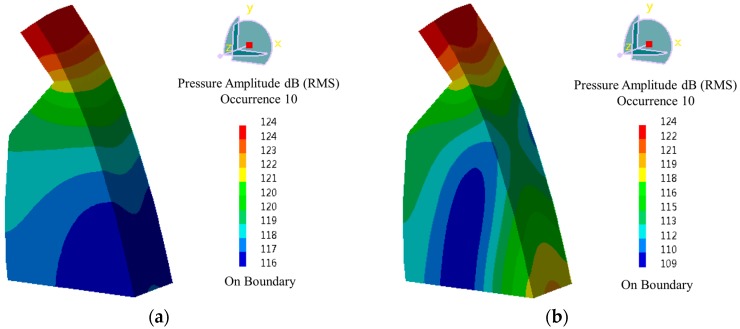

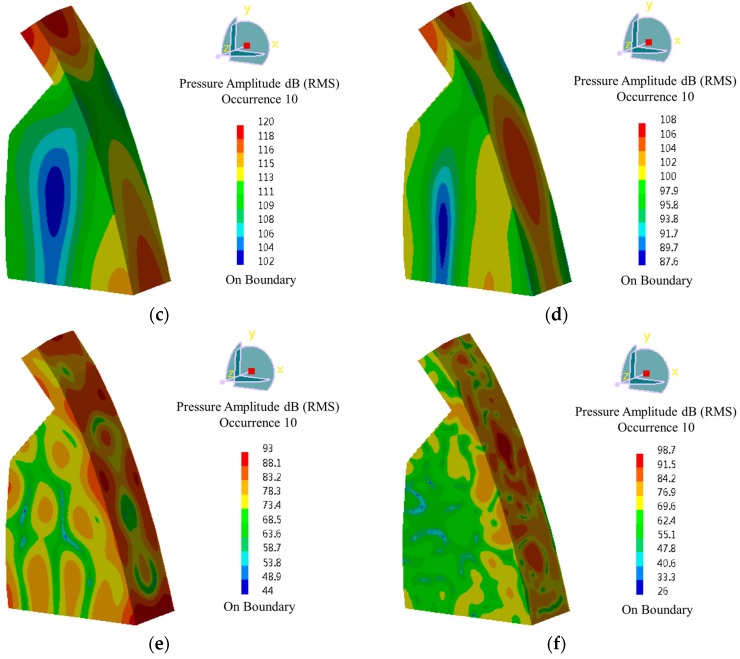
Contours of acoustic pressure of cavity under different frequency of Condition 6: (**a**) f = 0.55 Hz; (**b**) f = 1.50 Hz; (**c**) f = 2.25 Hz; (**d**) f = 3.00 Hz; (**e**) f = 7.00 Hz; (**f**) f = 15.00 Hz.

In this section, the LMS Virtual. Lab Acoustics software is adopted to analyze vibration response of the cavity. The boundary element model is formed through importing the finite element grid model into the acoustic module. Figure 18 shows an amplitude spectrum of the longitudinal flow velocity of 3# crest overflowing orifice. The amplitude spectrum can be loaded on the surface of continuous nappe jet of the cavity model as a boundary condition. The range of frequency response calculated is set as f = 0–20 Hz, Δf = 0.05 Hz. Figure 19 shows the contours of acoustic pressure under different exciting frequency.

As it can be seen in Figure 19, the distribution of acoustic pressure is simple in lower frequency and becomes increasingly complex in higher frequency band. The contours of cavity acoustic pressure are more similar to the cavity acoustic modal vibration mode when the frequency is lower. The amplitude of cavity acoustic pressure level reaches 124 dB when the frequency is 0.55 Hz. The pressure level reaches 124 dB when the frequency is 1.50 Hz, and it reaches 120 dB when the frequency is 2.25 Hz. The analyses above indicate that high-energy LFN is generated from the vibration of the cavity behind the nappe jets, which is excited by the turbulent fluctuation of the flow velocity.

**Figure 20:**
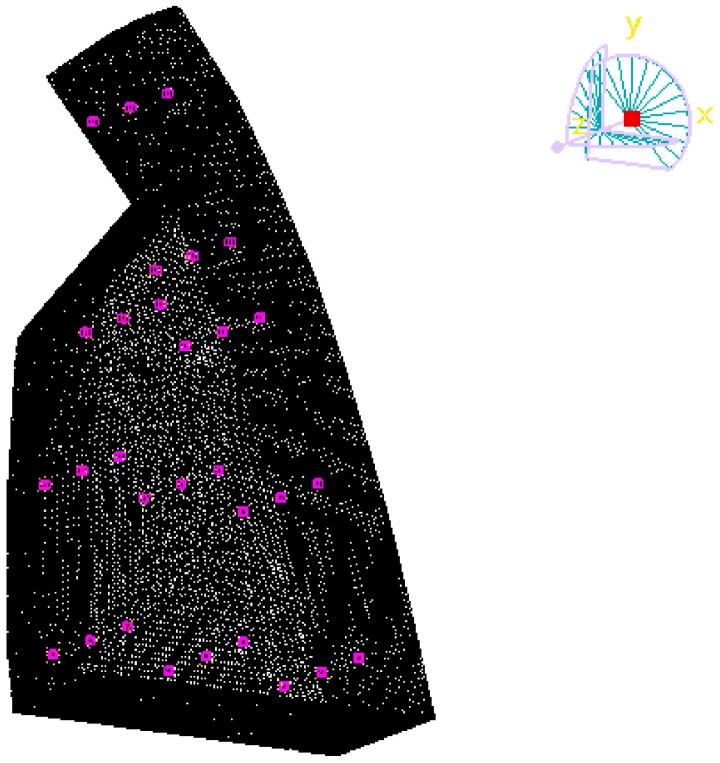
Layout of observation points of acoustic pressure in the cavity.

**Figure 21:**
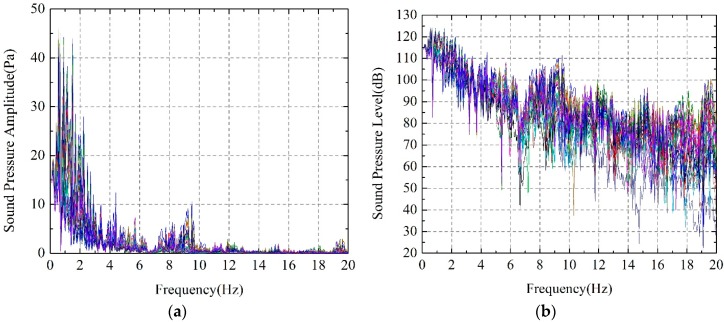
Frequency response functions of acoustic pressure of observation points: (**a**) Acoustic pressure; (**b**) Acoustic pressure level.

Furthermore, 30 prediction points have been set in different location of the cavity to evaluate the acoustic field of the cavity behind the nappe jets, as it can be seen in Figure 20. After acoustic simulation calculation, Figure 21 shows the frequency response function of acoustic pressure and acoustic pressure level.

**Figure 22:**
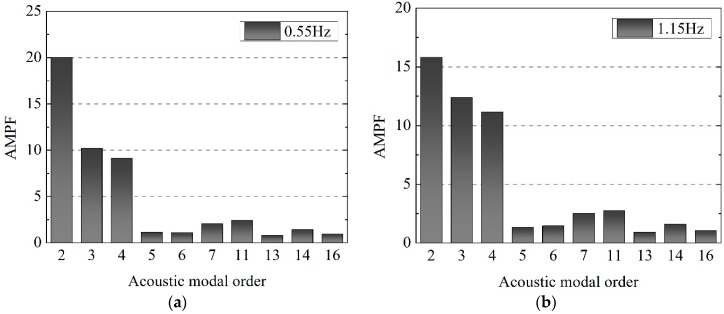

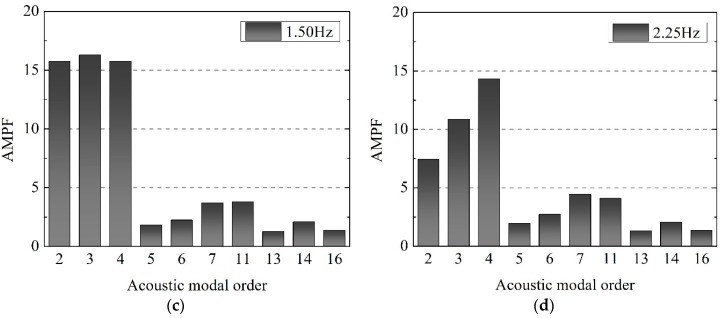
Acoustic modal participation factors of peak frequency of: (**a**) 0.55 Hz; (**b**) 1.15 Hz; (**c**) 1.50 Hz; (**d**) 2.25 Hz.

The frequency response functions of acoustic pressure and acoustic pressure level data of all these points has been overlapped in Figure 21. It can be seen that the acoustic pressure in the cavity suddenly increases in frequencies of 0.55 Hz, 1.15 Hz, 1.50 Hz and 2.25 Hz. The cavity resonance and strong noise can be excited because the modal frequencies of the cavity are close to the frequencies of the nappe jets vibration. Figure 22 shows the AMPF of these peak frequencies to determine the contribution of every frequency. For the acoustic response lower than 4 Hz, it is seen that the second, the third and the fourth order acoustic modal of cavity have lager participation factor, and their frequencies are 1.054 Hz, 1.676 Hz and 1.974 Hz. The results of the acoustic modal and acoustic field calculation indicate that the coupled vibration of nappe-cavity contributes not only to the acoustic power that within 0–1 Hz, but also to the power higher than 1 Hz, which explained the acoustic power with frequency within 1–4 Hz observed in prototype observation.

### 3.4. Analysis of Contribution Degree of Multi-Sources to LFN Energy

The multi-sources mechanisms of LFN generated in the flood discharge and energy dissipation of ski-jump type of a high dam have been revealed by the analyses above. In this section, the contributions of different mechanisms to the LFN energy of prototype will be calculated. The LFN energy consists of the energy caused by submerged jets in plunge pool and nappe-cavity coupled vibration. The equation can be expressed as:

(23) $${\bar{P}}_{\omega }^{2}+{\bar{P}}_{c}^{2}=\sum_{y}p_{\omega }^{2}+\sum_{c}p_{c}^{2}=P^{2},$$

where P is effective acoustic pressure of a point in prototype observation; ${\bar{P}}_{c}$ is the total effective acoustic pressure radiated from the nappe-cavity coupled vibration; ${\bar{P}}_{\omega }$ is the total effective acoustic pressure radiated from acoustic source region of the submerged jets in plunge pool, which can be calculated by substituting the numerical results into the model described in Section 2.2; $p_{\omega }$ is the contribution of the pressure of point sources in the area of the submerged jets; $p_{c}$ is the contribution of the pressure of point sources in the area of the nappe-cavity. Then a parameter, which describes the influence coefficient that submerged jets contributes to LFN energy, is defined as $IC_{1}={\bar{P}}_{\omega }/P$. The influence coefficient that nappe-cavity coupled vibration contributes to LFN energy is defined as $IC_{2}={\bar{P}}_{c}/P$. The influence coefficients of multi-sources of the LFN observed in prototypes can be calculated by Equation (23) and the data from prototype observation. The results have been presented in Table 6.

In Table 6, it can be seen that the submerged jets are the only acoustic source for the condition of discontinuous orifice opening, such as Conditions 2 and 8. As for the condition of continuous orifice opening, such as Conditions 5 and 6, both submerged jets and nappe-cavity coupled vibration contribute to LFN energy. The average impact factor of submerged jets is 0.619, while the average impact factor of nappe-cavity coupled vibration is 0.785. The contribution of nappe-cavity coupled vibration is a little higher. Table 7 shows the LFN strength prediction of some other points of Condition 6 by using the result of influence coefficient above. The theoretical prediction of the LFN pressure ($P_{T}$) is basically consistent with the result of prototype observation ($P_{P}$), and the relative error is lower than 2.1%. Therefore, the strength of LFN generated by flood discharging and energy dissipation of the ski-jump type of a high dam can be estimated through the analyses above.

## 4. Conclusions

In this study, the results of prototype observation indicate that LFN is generated during the flood discharge and energy dissipation of a high dam with a ski-jump type spillway. The LFN intensity attenuates at different rates in different distances away from the dam. The LFN observed in the conditions with adjacent orifices opening has wider energy distribution and several peak frequencies within 0–4 Hz, which indicates that multi-sources generation mechanisms make contribution to induce the LFN during the flood discharge of Jinping First Stage Hydropower Station.

The generation mechanism for LFN induced by submerged jets has been studied basing on the numerical simulation of the turbulent flow. The VFF data is extracted from the numerical results and analyzed in time and frequency domain. The comparison between the data of VFF and prototype observation indicates that the LFN induced by submerged jets only contribute to 0–1 Hz LFN energy on site, and the strong shear layers around high-velocity submerged jets and wall jet development area are the main acoustic source regions of LFN in plunge pool.

The generation mechanism of LFN induced by nappe-cavity coupled vibration is analyzed by FEM/BEM method. The results of acoustic modal and acoustic field calculation reveals that the coupled vibration of nappe-cavity contributes not only to the acoustic power that within 0–1 Hz, but also to the power higher than 1 Hz, which explained the acoustic power with frequency within 1–4 Hz observed in prototype observation.

The results in this study suggest that both kinds of LFN generation mechanisms have approximate contribution degrees, while the contribution of nappe-cavity coupled vibration is a little higher. We conclude that two kinds of mechanisms contribute to the LFN induced by flood discharge and energy dissipation of the ski-jump type spillway of a high dam. One is the mechanism of vortex of submerged jets in plunge pool, and the other is the mechanism of nappe-cavity coupled vibration. In additions, the conditions with more adjacent orifices opening uniformly have been found effective to reduce the LFN intensity according to the results of prototype observation. Therefore, we suggest more adjacent orifices to be opened uniformly in a certain discharge capacity during the operation period of the dam. Nevertheless, the mathematical model to exactly calculate the LFN pressure has not been proposed. Further investigations should focus on the quantitative forecast of the LFN induced by flood discharge and energy dissipation of the ski-jump type spillway of a high dam.

## Figures and Tables

**Table 1:** Observed discharge conditions *.

No.	Q (m^3^/s)	Water Level Elevation (m)		Bottom Dscharge Orifice	Crest Overflowing Orifice
		Upstream	Downstream		
1	0	/	/	/	/
2	1090	1880.00	1646.61	3#	/
3	2080	1880.00	1647.53	2#, 4#	/
4	4360	1880.00	1650.08	1#, 2#, 4#, 5#	/
5	5450	1880.00	1651.98	1#, 2#, 3#, 4#, 5#	/
6	3840	1880.00	1650.02	/	1#2#3#4#
7	4100	1880.00	1651.26	2#, 4#	2#, 3#
8	5190	1880.00	1651.69	2#, 3#, 4#	2#, 3#

* Q is the total discharge through the dam orifices. The discharge orifice’s number is named from the left bank to the right bank.

**Table 2:** Correlation between the LFN intensity, P/Q and Q.

Condition No.	Q (m^3^/s)	T7		T9		T11	
		P (Pa)	P/Q (Pa/(m^3^/s))	P (Pa)	P/Q (Pa/(m^3^/s))	P (Pa)	P/Q (Pa/(m^3^/s))
2	1090	7.164	0.00657	6.404	0.00588	4.911	0.00451
3	2080	8.942	0.00430	7.155	0.00344	5.255	0.00253
4	4360	10.561	0.00242	8.893	0.00204	6.460	0.00148
5	5450	10.754	0.00197	8.971	0.00165	6.662	0.00122
6	3840	8.007	0.00209	6.435	0.00168	4.467	0.00116
7	4100	11.251	0.00274	8.726	0.00213	6.599	0.00161
8	5190	11.993	0.00231	10.249	0.00197	8.301	0.00160

**Table 3:** Comparison between theoretical and numerical results.

Condition No.	Analysis Items of Nappe	Numerical Results	Theoretical Results	Relative Error (%)
2	Trajectory distance of 3# bottom discharge orifice (m)	85.634	88.657	3.410
	Initial water-entry velocity of 3# bottom discharge orifice (m/s)	58.991	60.930	3.182
5	Trajectory distance of 3# bottom discharge orifice (m)	86.441	88.243	2.042
	Initial water-entry velocity of 3# bottom discharge orifice (m/s)	59.026	60.593	2.586
6	Trajectory distance of 3# crest overflowing orifice (m)	87.526	89.181	1.856
	Initial water-entry velocity of 3# crest overflowing orifice (m/s)	63.832	65.375	2.360
8	Trajectory distance of 3# crest overflowing orifice (m)	84.079	86.024	2.261
	Initial water-entry velocity of 3# crest overflowing orifice (m/s)	63.759	65.628	2.848
	Trajectory distance of 3# bottom discharge orifice (m)	84.172	86.265	2.426
	Initial water-entry velocity of 3# bottom discharge orifice (m/s)	58.133	60.121	3.307

**Table 4:** Analyses of vorticity monitoring data.

Condition 2				Condition 6			
Monitoring Point	Time-Averaged Value (s^−1^)	RMS (s^−1^)	Dominant Frequency (Hz)	Monitoring Point	Time-Averaged Value (s^−1^)	RMS (s^−1^)	Dominant Frequency (Hz)
B1	24.601	0.920	0.227	B1	27.876	3.175	0.196
B2	26.961	11.406	0.745	B2	24.723	3.756	0.471
B3	1.749	0.878	0.318	B3	2.843	1.672	0.269
B4	17.823	1.333	0.182	B4	19.889	2.825	0.318
B5	20.502	4.764	0.364	B5	24.866	6.208	0.567
B6	12.744	2.448	0.182	B6	16.235	1.505	0.269
B7	5.599	2.779	0.427	B7	22.844	5.334	0.710
B8	0.249	0.032	/	B8	12.099	1.205	0.196
B9	7.764	3.411	0.436	B9	20.964	8.566	0.441
B10	3.181	0.974	0.182	B10	8.010	1.513	0.196
				B11	19.654	12.389	0.563
				B12	5.555	1.794	0.392
				B13	8.399	6.021	0.514

**Table 5:** Acoustic modal frequency of the cavity of Condition 6.

**Order No.**	1	2	3	4	5	6	7	8	9	10
**Modal Frequency**	0	1.054	1.676	1.974	2.633	2.657	2.797	2.900	3.169	3.318

**Table 6:** Influence coefficient of multi vibration source of LFN.

Condition No.	Observation Point	*P* (Pa)	${\bar{P}}_{\omega }$ (Pa)	*IC*_1_	${\bar{P}}_{c}$ (Pa)	*IC*_2_
2	T9	6.404	6.995	≡1	/	/
	T11	4.911	5.151	≡1	/	/
5	T9	8.971	5.722	0.638	6.910	0.770
	T11	6.662	4.109	0.617	5.244	0.787
6	T9	6.435	3.865	0.601	5.145	0.799
	T11	4.467	2.775	0.621	3.500	0.784
8	T9	10.249	10.620	≡1	/	/
	T11	8.301	9.172	≡1	/	/

**Table 7:** Verification of influence coefficients of multi vibration sources of LFN.

Condition No.	Observation Point	*IC*_1_	*IC*_2_	${\bar{P}}_{\omega }$ (Pa)	${\bar{P}}_{c}$ (Pa)	*P_T_* (Pa)	*P_P_* (Pa)	Relative Error (%)
6	T5	0.619	0.785	6.989	8.863	11.291	11.532	2.091
	T12			1.734	2.199	2.801	2.839	1.339
